# A Synthetic Ultra-Wideband Transceiver for Millimeter-Wave Imaging Applications †

**DOI:** 10.3390/mi14112031

**Published:** 2023-10-31

**Authors:** Amir Mirbeik, Laleh Najafizadeh, Negar Ebadi

**Affiliations:** 1 RadioSight LLC, Hoboken, NJ 07030, USA; 2 Department of Electrical and Computer Engineering, Rutgers University, Piscataway, NJ 08854, USA; 3 Department of Electrical and Computer Engineering, Stevens Institute of Technology, Hoboken, NJ 07030, USA; 4 Stanford University School of Medicine, Stanford, CA 94305, USA

**Keywords:** millimeter-wave imaging, transceiver, circuit, biomedical imaging, ultra-wideband, CMOS

## Abstract

In this work, we present a transceiver front-end in SiGe BiCMOS technology that can provide an ultra-wide bandwidth of 100 GHz at millimeter-wave frequencies. The front-end utilizes an innovative arrangement to efficiently distribute broadband-generated pulses and coherently combine received pulses with minimal loss. This leads to the realization of a fully integrated ultra-high-resolution imaging chip for biomedical applications. We realized an ultra-wide imaging band-width of 100 GHz via the integration of two adjacent disjointed frequency sub-bands of 10–50 GHz and 50–110 GHz. The transceiver front-end is capable of both transmit (TX) and receive (RX) operations. This is a crucial component for a system that can be expanded by repeating a single unit cell in both the horizontal and vertical directions. The imaging elements were designed and fabricated in Global Foundry 130-nm SiGe 8XP process technology.

## 1. Introduction

Millimeter-wave imaging (MMWI) systems find widespread use in both military and commercial contexts, serving various purposes, such as identifying concealed dangers, enhancing surveillance and security measures, and facilitating non-destructive testing [1,2,3,4,5,6]. A recent investigation conducted by our team revealed notable distinctions in the dielectric characteristics between healthy skin and two prevalent forms of skin cancer, basal cell carcinoma and squamous cell carcinoma, in millimeter-wave frequencies [7]. This research comprised the acquisition of 101 skin tissue specimens from individuals with skin cancer, and the measurement of reflection coefficients was carried out utilizing a network analyzer across a frequency spectrum ranging from 0.5 GHz to 50 GHz. The statistically significant variations in dielectric properties between cancerous and healthy skin were particularly pronounced for squamous cell carcinoma. These findings suggested that millimeter waves might serve as valuable resources for the diagnosis of skin cancer.

**Figure 1:**
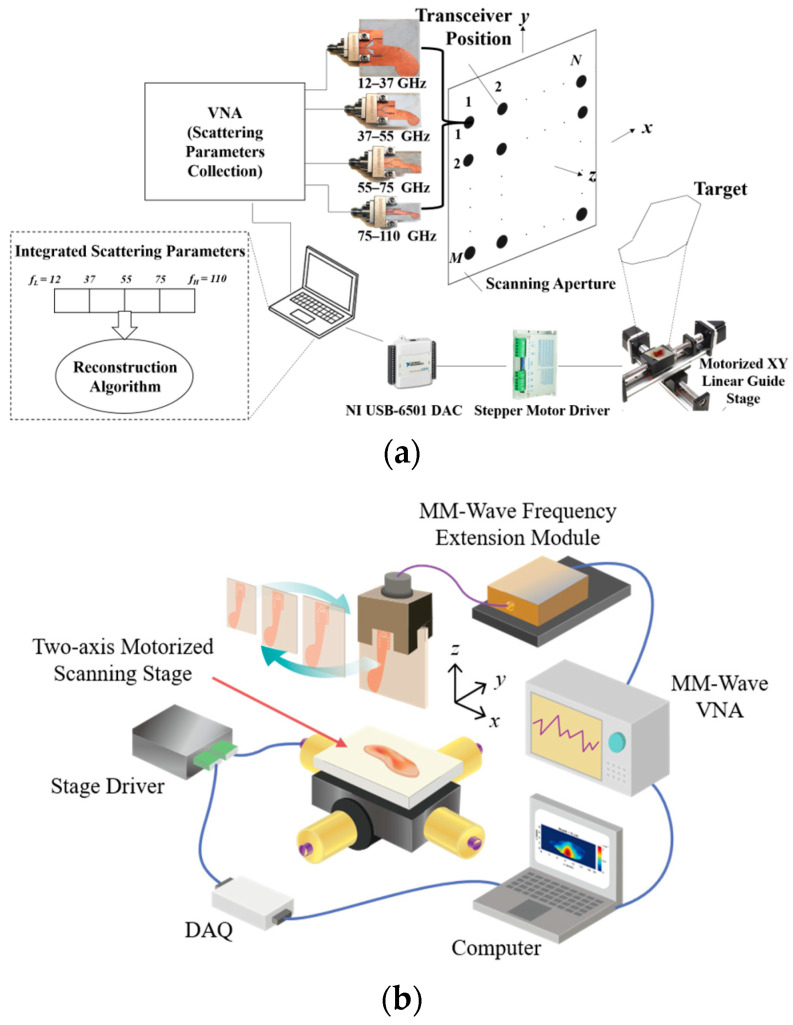
(**a**) The synthetic ultra-wideband millimeter-wave imaging (MMWI) approach. (**b**) The MMWI system for skin cancer imaging, realizing an overall synthetic bandwidth of 98 GHz. At each scanning step, four sub-band antennas are successively placed in front of the target to transmit their signals in their respective sub-bands and record the backscattered signals. The scanning process is automatically performed using a motorized stage in conjunction with two drivers, a computer with LabVIEW, and a data acquisition (DAQ) device, which generates the digital control signals from the computer [9,10].

While MMWI systems offer the potential to detect cancer due to providing high image contrasts, they are not typically used for this purpose due to their inadequate resolutions for imaging biological tissues. In order to overcome this constraint, our research group suggested a new imaging method termed “Synthetic Ultra-Wideband Millimeter-wave Imaging”, which greatly improves the resolution of MMWI systems [8]. As shown in Figure 1a, this approach involves combining several neighboring imaging sub-bands to create an ultra-wide imaging bandwidth. Each sub-band incorporates an element exclusively designed for its designated frequency range. Positioned facing the target across the scanning aperture, these sub-bands transmit signals and capture the backscattered reflections. The captured signals are combined through a signal combination algorithm to create a synthesized, ultra-wideband signal.

In a separate investigation, we created a proof-of-concept system that boasts a remarkably broad bandwidth of 98 GHz through the use of the above-mentioned MMWI technique [9]. The design of this system, which merges four adjacent sub-bands to form an ultra-wideband signal, is shown in Figure 1b. A measurement platform that can scan tissues across a rectangular aperture plane was created to automate the process. A novel imaging algorithm was also implemented to analyze the signals and generate a representation of cancerous tissue in the frequency domain. The image is created utilizing a reflectivity function, defined as the ratio of the echoed fields to the incident signals.

Although the ex vivo imaging setup was sufficient for initial experiments, it was not practical for in vivo imaging due to the lengthy measurement time. To tackle this concern, the quantity of sub-band antennas was decreased to two, resulting in a significantly accelerated imaging process of under 20 s. This advancement gave rise to the creation of a real-time imaging system for the in vivo diagnosis of skin cancer [10]. This system underwent testing on 136 skin lesions, both benign and malignant, sourced from seventy-one patients. Lesions were categorized utilizing 3D principal component analysis and five distinct classifiers. The findings indicated that the most effective classification was attained using five principal components, coupled with a multi-layer perceptron model, resulting in a sensitivity of 97% and specificity of 98% [10]. These results illustrated that real-time MMWI could effectively differentiate between benign and cancerous skin lesions, positioning it as a promising diagnostic tool. The institutional review board (IRB) at the Hackensack University Medical Center (Protocol Pro 2019-0219) approved this research. The experimental protocols adhered to the guidelines set by the IRB. Prior to conducting measurements, each patient gave written informed consent [10].

## 2. Problem Statement

Utilizing the expansive bandwidths found in millimeter-wave frequencies, imaging and communication systems can attain imaging with high resolution and facilitate rapid communication [11]. In the biomedical field, several research groups have presented millimeter-wave imaging systems based on silicon in the last decade. These systems aim to attain high image resolutions, and rapid times of acquisition [12,13,14]. However, before this study, there was no imaging chip capable of providing the resolution adequate for visualizing biological tissues. In the context of wideband imaging, resolution is linked to the system’s bandwidth [14]. It has been suggested that a millimeter-wave bandwidth of around 100 GHz is required to attain satisfactory resolutions for skin images [8].

In [15], we presented three pulse generators that were integrated and designed to jointly produce a bandwidth of 100 GHz in the millimeter-wave spectrum. These pulse generators release signals that span frequencies of 10–40 GHz, 40–75 GHz, and 75–110 GHz. This advancement enabled the development of a fully integrated ultra-high-resolution imaging chip using the synthetic ultra-wideband MMWI technique. 

Here, we crafted a front-end of a transceiver that incorporates the above-mentioned technique to achieve the realization of a fully integrated ultra-high-resolution imaging chip for biomedical applications. As will be shown, we achieve an ultra-wide imaging bandwidth of 100 GHz through the integration of two adjacent disjointed frequency sub-bands of 10–50 GHz and 50–110 GHz. The imaging components were designed and manufactured using Global Foundry’s 130 nm silicon germanium (SiGe) process technology. This paper is an extension of a paper published in 2021 IEEE International Symposium on Antennas and Propagation and USNC-URSI Radio Science Meeting (APS/URSI) in Singapore [15].

**Figure 2:**
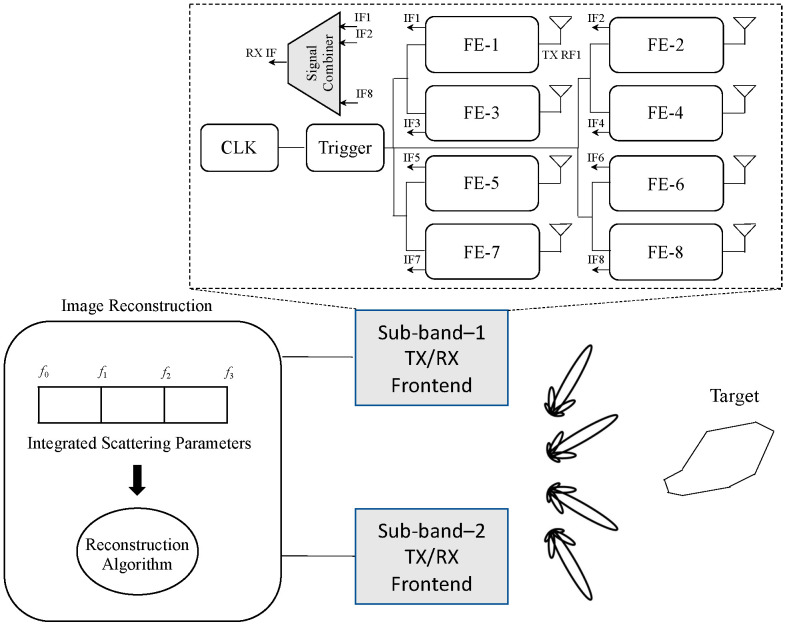
Schematic of the synthetic ultra-wideband millimeter-wave imaging technique. Three transceivers operating in distinct sub-bands transmit signals and receive the corresponding backscattered signals.

The representation of the ultra-wideband imaging chip is illustrated in Figure 2. As depicted in the diagram, each sub-band is equipped with an independent imaging transceiver that exclusively functions within that particular sub-band. Leveraging the transceivers with embedded phased-array capability, the antenna beams of the sub-band could be electronically directed to scan the complete region of interest. The received intermediate frequency (IF) signals undergo filtration and are directed to the image reconstruction module. Within this module, an integrated signal is synthesized across the entire bandwidth, resulting in the formation of a three-dimensional (3D) image.

The inset in Figure 2 exhibits the representation of each transceiver, comprising eight components. Each component is furnished with a delay line that regulates the timings of radiation for the coherent synthesis of wideband signals. We utilized on-chip antennas to mitigate loss in power and distortion in phase that may arise from connections to antennas located off the chip. Furthermore, every antenna served the dual purpose of both radiating and receiving waves towards and from the target. 

The subsequent sections delve into a discussion on the system’s architecture and provide details into the primary components of the front-end, consisting of a low-noise amplifier (LNA), a multiplier, a transmit/receive (T/R) switch, a pulse generator, and on-chip antennas. Following this, we present the results of the characterization of the simulated and fabricated chips.

## 3. System Design

The synthetic-aperture radar (SAR) technique is frequently employed in millimeter-wave imaging studies to generate 3D reconstructions of objects. However, scanning across a two-dimensional (2D) aperture plane results in a larger system size, which is impractical for silicon-based systems. In this study, electronic beamforming was utilized to scan the objects instead. The incorporation of beamforming capabilities enhances the signal-to-noise ratio (SNR) of the system.

The transceiver front-end was designed for both transmit and receive operations, as shown in Figure 2. This is a crucial component of the system that can be expanded by repeating a single unit cell in both the horizontal and vertical directions.

**Figure 3:**
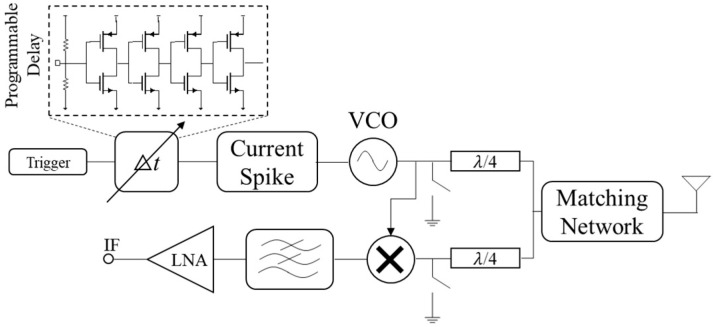
Schematic of an array element. The digital trigger undergoes processing through a programmable delay generator. The element is additionally equipped with a pulse generator, a multiplier, a low-noise amplifier (LNA), a transmit/receive (T/R) switch, and an on-chip antenna.

Figure 3 displays the schematic of an array element’s front-end. It comprises an LNA, a mixer, a T/R switch, a pulse generator, and on-chip antennas integrated into silicon. Distinct circuits must be developed for each component to match the specifications of the two frequency sub-bands, namely 10–50 GHz and 50–110 GHz.

In this work, a pulse-radiating approach was adopted, as it provides a wider relative bandwidth and higher image resolution compared to continuous-wave radiators [16]. A delay generator was used to regulate the signal’s timing according to the supply voltage. As demonstrated in Figure 3, the trigger undergoes processing in a delay generator, consisting of a sequence of inverter stages that manage the signal’s timing in accordance with the supply voltage. In transmit mode, the pulse produced via the pulse generator is delayed and transmitted through an antenna. During reception mode, the front-end captures the backscattered reflection and combines it with a delayed version of the signal.

The description of the design for each essential building block is provided in the following sections.

### 3.1. Pulse Generator

The designs presented in this study utilized a system architecture based on the pulse voltage-controlled oscillator (VCO) suggested in our previous work [15]. The pulse generators transform the input signal into generated pulses with precise timing, essential for the coherent combination of pulses. An asymmetrical cross-coupled configuration was employed to reduce the timing jitter of the radiated pulses and achieve a coherent combination. This led to a notable decrease in power usage, system intricacy, and chip space. The pulse generators were designed to produce pulses having full-width-at-half-maximums (FWHMs) of 14 ps and 10 ps for the two sub-bands of 10–50 and 50–110 GHz, respectively.

**Figure 4:**
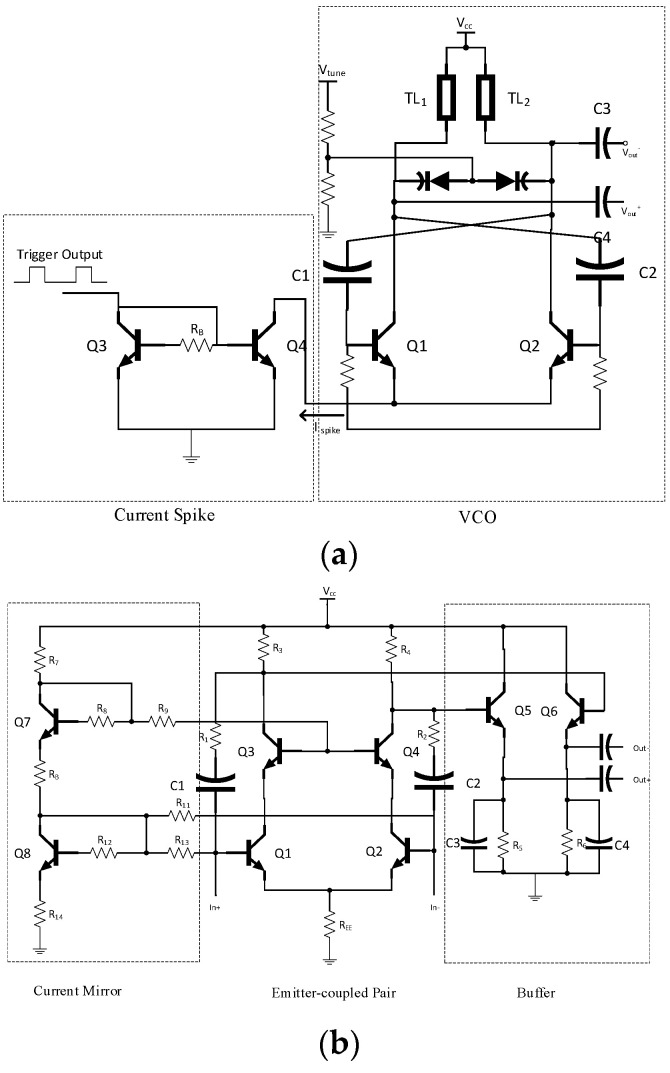

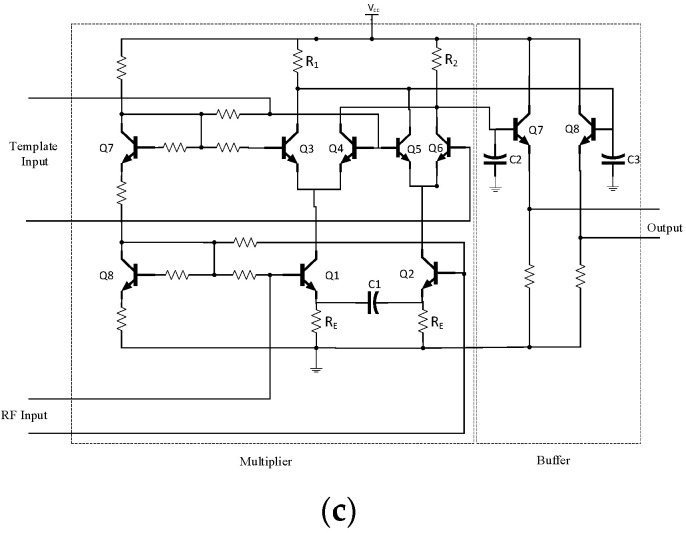
The schematic of (**a**) the pulse generator in this work, consisting of a current spike and a pulse VCO with asymmetric cross-coupled topology; (**b**) the LNA in this work, consisting of an emitter-coupled pair followed by two emitter follower stages as buffers; and (**c**) the wideband correlator with a multiplier, a low-pass filter, and a differential buffer.

Figure 4a shows the complete schematic of the pulse generator, consisting of a cur-rent spike and an asymmetrical cross-coupled pulse VCO configuration. When the peak current is sufficiently elevated to induce negative resistance at the collectors of Q1 and Q2, the VCO is turned on. A Schmitt trigger supplied the current spike circuit.

### 3.2. On-Chip Antennas

In order to minimize feedline radiation, a broadband slot differential antenna was considered, employing two excitations with even amplitudes and opposing phases. When crafting on-chip antennas, a potential issue is posed by the surface waves, since they may diminish antenna radiation efficiency. One approach to address this concern is the utilization of a silicon lens [17], but this may constrain the antenna’s field of view. Another strategy involves optimizing the silicon substrate’s thickness to enhance radiation efficiency. The antennas were specifically devised to emit through the substrate, considering the potential impact of wire bonds on topside radiation. Due to spatial constraints, the on-chip antennas were confined to a maximum dimension of 100 μm. The electromagnetic simulator ANSYS HFSS was employed for the simulation and optimization of the on-chip antennas.

### 3.3. Low-Noise Amplifier

We used a fully differential structure for the two sub-band low-noise amplifiers. The designed LNAs utilize an emitter-coupled design, exhibiting resistive feedback [18]. They have small die areas by avoiding large on-chip curved inductors. Figure 4b displays the schematic of the LNA. It consists of emitter-coupled transistors followed by buffer stages [18]. A current mirror was employed to set the bias. The output and input were in a differential configuration. The LNA will be linked to the slot antenna and will directly supply a Gilbert cell mixer. The emitter-coupled transistors were symmetric by incorporating the same transistors and passive elements in both branches.

### 3.4. Mixer

Figure 4c displays the diagram of the wideband correlator. The mixer’s core features a cross-coupled Gilbert cell configuration, incorporating two parallel stages in differential mode. This configuration is accompanied by a differential buffer. Biasing for the Gilbert cell mixer was achieved through a loaded current mirror. The employment of C1, along with *R*_E_ in the emitter, creates a capacitive resistive shunt load, which compensates for gain drop at higher frequencies. This compensation ensures a group delay and gain uniformity across the desired frequency range. The low-pass filters were constituted by the Gilbert cell’s load resistors (*R*_1_ and *R*_2_) in conjunction with the buffers’ shunt capacitors (C2 and C3). These filters served to identify the envelope of the mixed signals. The maximum average power consumption of the sub-band correlators was 30 mW.

## 4. Simulation Results

**Figure 5:**
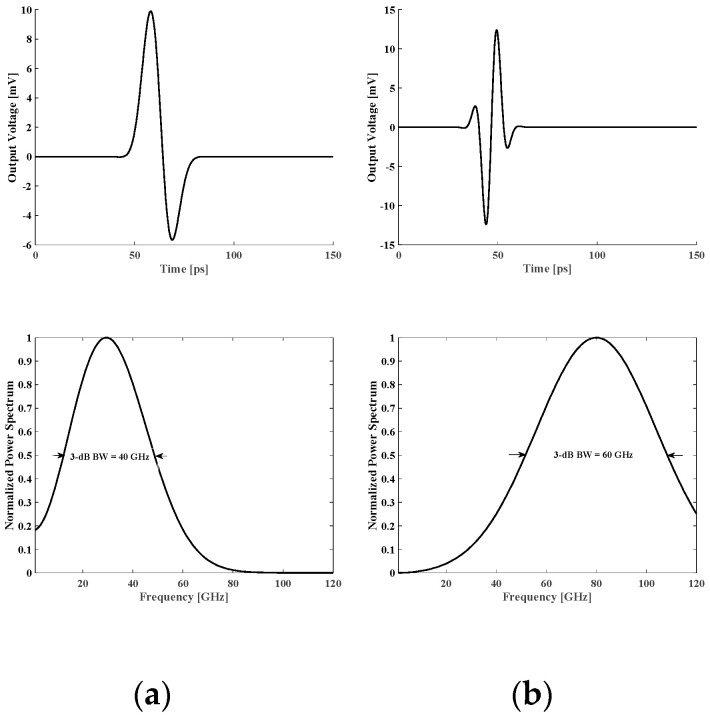
Simulated output pulses (top) and normalized power spectrums (bottom) of the (**a**) 10–50 GHz and (**b**) 50–110 GHz pulse generators.

Figure 5 (top) shows the simulated pulses generated via the two sub-band pulse generators. The normalized power spectrums are demonstrated in the bottom row of Figure 5. The simulated center frequencies were close to the desired values (25 GHz and 80 GHz) and the 3 dB bandwidths were approximately 40 GHz and 60 GHz.

**Figure 6:**
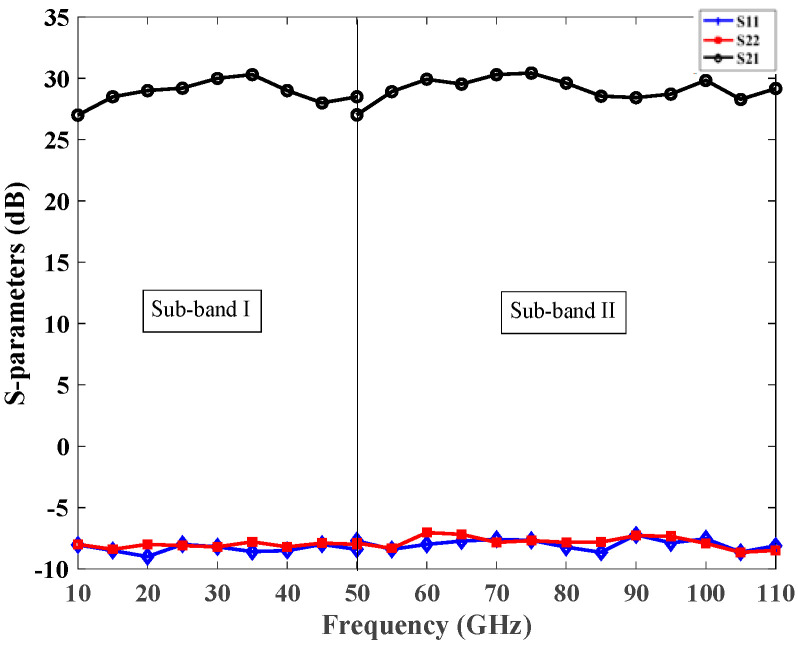
Simulated performance of the LNAs.

The simulated performance of the sub-band LNAs is demonstrated in Figure 6. In the front-end of the transceiver, the output of the switch was connected to the matching network input of the LNA (Figure 3). Consequently, the design of the LNA-matching network was tailored to the output of the switch instead of for a resistance of 50 Ω. The simulated return loss (referred to 50 Ω) was higher than 10 dB. This was due to the different impedance that the input of the LNA experiences in the detached scenario in contrast to the situation with the switch in place.

**Figure 7:**
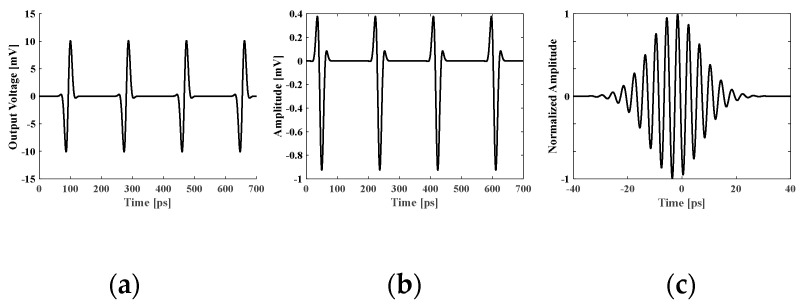
(**a**) Simulated output pulse; (**b**) simulated received pulse directly after the antenna; and (**c**) normalized cross-correlation of the received pulse with template pulses.

To showcase the transceiver’s suitability for imaging purposes, a pulse train with a 2 GHz repetition rate was fed into the transceiver corresponding to the first sub-band (10–50 GHz). The simulated pulse train can be seen in Figure 7a. The received signal is shown in Figure 7b. The antenna responses were included in the received signal. To characterize the correlation performance, the output of the receiver was linked to a low-pass filter featuring a 20 KHz 3 dB corner frequency. The simulated cross-correlation of the reference pulses with the received pulses is demonstrated in Figure 7c. A key limitation of the suggested tiled sub-band front-end approach was the front-end size. All sub-band front ends must be integrated in an area smaller than 1.5 mm by 1.5 mm to guarantee the space of 1.6 mm (~*λ*/2) between sub-band front ends, while providing ample space for sampling and signal merging and distribution among the tiles. The compact imaging front-end designed in this work allows for the creation of scalable arrays that embrace both transmit and receive operations.

**Figure 8:**
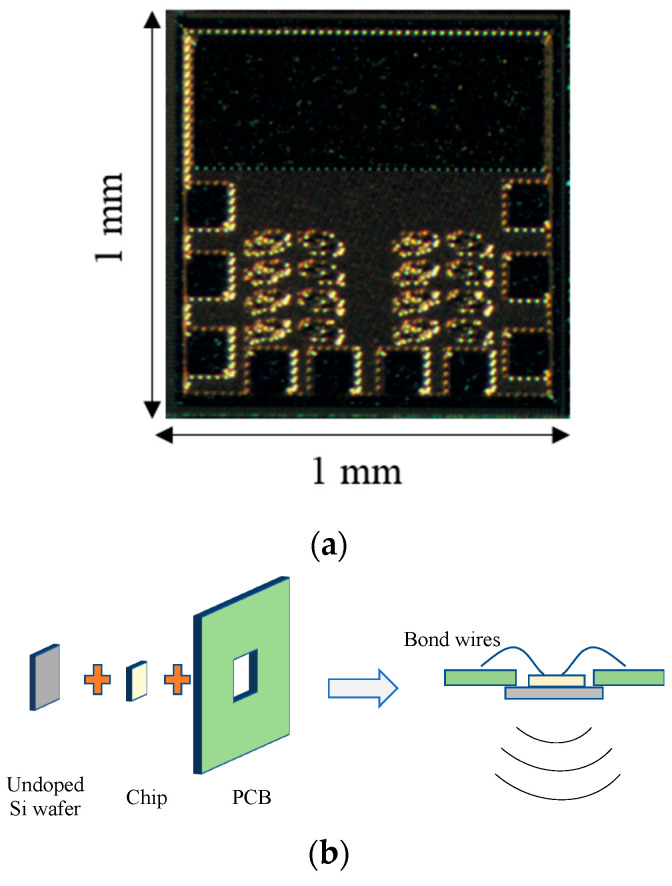
(**a**) Chip micrograph and (**b**) chip assembly.

The imaging chip was fabricated in a 130 nm BiCMOS (8XP) technology process, with a cutoff frequency (*f*T) of 200 GHz and a maximum oscillation frequency (*f*max) of 265 GHz. The fabricated chip micrograph is shown in Figure 8a. It covers an area of 1 mm × 1 mm. It was affixed to the backside of a Roger PCB, featuring a hole to allow radiation through the air. Wire bonding was employed to establish connections between the PCB and chip pads, as shown in Figure 8b.

**Figure 9:**
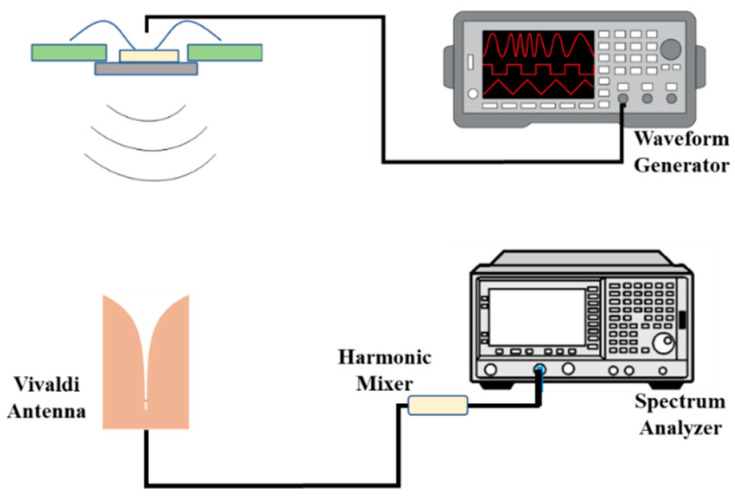
Measurement setup for characterization of the fabricated chip.

The imager chip is characterized in the frequency domain, as demonstrated in Figure 9. A pulse generator was used to create the trigger signal. A substrate integrated waveguide (SIW)-based Vivaldi antenna, which we had previously developed [8], was used as a receiving antenna. A harmonic mixer was used to down-convert the high frequencies of the received signal into low-frequency components at which the spectrum analyzer operates. Due to the limited frequency range of the harmonic mixer (75 GHz–110 GHz), the frequency domain characterization was only performed for the second sub-band (50 GHz–110 GHz).

**Figure 10:**
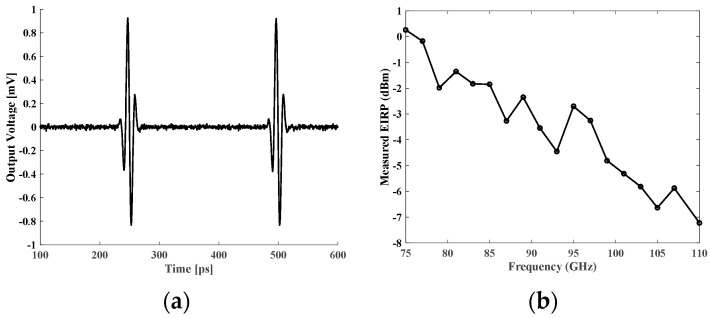
(**a**) Measured pulse waveform for the second sub-band with a 13 ps FWHM pulse width. (**b**) Measured EIRP spectrum for the second sub-band (50–110 GHz).

Figure 10a presents the measured pulse waveform for the second sub-band, with a FWHM pulse width of 13 ps. Its average equivalent isotropically radiated power (EIRP) frequency spectrum is shown in Figure 10b. As shown in this figure, the radiated pulse holds a 0.3 dBm average EIRP at 75 GHz and a 5 dB bandwidth of nearly 50 GHz. The difference between the measured and simulated center frequencies (75 GHz and 80 GHz, respectively) arises from the impacts of package PCB and variations in the manufacturing process. The chip average power consumption was 194 mW.

The above-mentioned characterizations confirm that the proposed imaging chip is capable of transmitting and receiving ultra-wideband signals at millimeter-wave frequencies with low power consumption and high precision. Further evaluations will include radiation pattern measurements.

## 5. Conclusions and Future Plans

Expanding upon our prior research [16], we developed a transceiver front-end that incorporates the synthetic ultra-wideband millimeter-wave imaging technique. This advancement aims to achieve a fully integrated, ultra-high-resolution imaging chip tailored for biomedical applications. Through the integration of two contiguous, yet distinct, frequency sub-bands (10–50 GHz and 50–110 GHz), we achieved an extensive imaging bandwidth of 100 GHz. The imaging components were meticulously designed and manufactured using the Global Foundry 130 nm SiGe technology. As discussed in Section 1, the imaging chip developed in this work promotes a dual-mode operation of receivers and transmitters. This ensures enhanced consistency, reduced system power consumption, and better system integration. A few characteristics of the designed imaging chip, specifically on the on-chip antennas’ side, will be improved in the future for having a more efficient imager. Various on-chip antenna configurations have been explored in the existing literature to enhance their performance in response to potential challenges within the chip surroundings [19,20,21,22,23]. These will be evaluated, and the best solution for our application will be selected.

**Figure 11:**
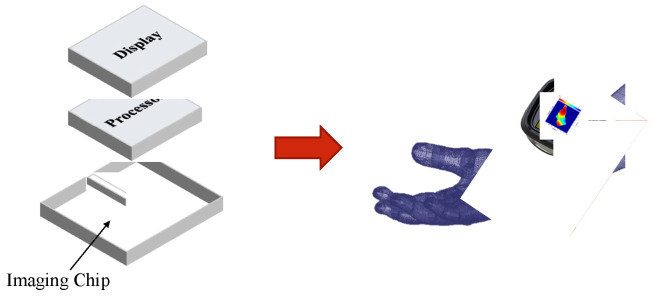
The rendered image of the compact imaging device.

In the future, we aim to assemble a prototype handheld imager comprising the im-aging chip developed in this work. The imager will also include a digital signal processor (DSP) to measure, control, and process continuous signals in the front-end module. The DSP will also execute the image reconstruction algorithm. Figure 11 shows the rendered image of the compact, handheld imaging device we envision. A display connected to the handheld housing and the processor, a switch connected to the processor, and a recharge-able power source connected to the handheld housing are also other components of the device.

## Data Availability

The datasets used and/or analyzed during the current study are available from the corresponding author on reasonable request.
